# Transcriptomic analysis comparing stay-green and senescent *Sorghum bicolor* lines identifies a role for proline biosynthesis in the stay-green trait

**DOI:** 10.1093/jxb/erv405

**Published:** 2015-08-28

**Authors:** Stephanie M. Johnson, Ian Cummins, Fei Ling Lim, Antoni R. Slabas, Marc R. Knight

**Affiliations:** ^1^Durham Centre for Crop Improvement Technology, School of Biological and Biomedical Sciences, Durham University, South Road, Durham DH1 3LE, UK; ^2^Unilever, Colworth Science Park, Sharnbrook, Bedford, MK44 1LQ, UK

**Keywords:** Drought, microarray, proline, sorghum, stay-green.

## Abstract

*Sorghum bicolor* is an important cereal crop grown on the arid and semi-arid regions of >98 different countries. These regions are such that this crop is often subjected to low water conditions, which can compromise yields. Stay-green sorghum plants are able to retain green leaf area for longer under drought conditions and as such have higher yields than their senescent counterparts. However, the molecular and physiological basis of this drought tolerance is yet to be fully understood. Here, a transcriptomic approach was used to compare gene expression between stay-green (B35) and senescent (R16) sorghum varieties. Ontological analysis of the differentially expressed transcripts identified an enrichment of genes involved with the ‘response to osmotic stress’ Gene Ontology (GO) category. In particular, delta1-pyrroline-5-carboxylate synthase 2 (*P5CS2*) was highly expressed in the stay-green line compared with the senescent line, and this high expression was correlated with higher proline levels. Comparisons of the differentially expressed genes with those that lie in known stay-green qualitative trait loci (QTLs) revealed that *P5CS2* lies within the Stg1 QTL. Polymorphisms in known *cis*-elements were identified in the putative promoter region of *P5CS2* and these could be responsible for the differences in the expression of this gene. This study provides greater insight into the stay-green trait in sorghum. This will be greatly beneficial not only to improve our understanding of drought tolerance mechanisms in sorghum, but also to facilitate the improvement of future sorghum cultivars by marker-assisted selection (MAS).

## Highlight

Genes that are differentially expressed between stay-green and senescent sorghum lines were identified. Analysis of these genes suggests a role for *P5CS2* in conferring the stay-green trait in sorghum.

## Introduction

Food security is a major challenge facing society today, with the global demand for food being expected to increase by up to 70% by 2050 (UNESCO, 2012). This increase in demand for food comes at a time when fresh water supplies are becoming more limited, meaning that many crops are grown under conditions of low water availability (Foley *et al.*, 2011). Consequently, drought stress is thought to be the biggest cause of crop yield reduction, particularly in the arid and semi-arid tropics (Boyer, 1982; Passioura, 2007). There is therefore an urgent requirement to improve our understanding of how crops are able to tolerate conditions of low water availability in order to help limit the problems associated with drought in the future.

The mechanisms of drought tolerance are complex, and the response of the plant depends upon the growth stage and the severity of the stress encountered (Rosenow *et al.*, 1983). With regard to plant morphology, an increased root depth and a thick waxy leaf cuticle can help plants to extract and retain more water, respectively. Physiological adaptations can allow for the control of water loss by transpiration via the stomata, and biochemical adaptations can act both to delay and to reduce the effects of the drought stress (Chaves *et al.*, 2003). One such biochemical mechanism is the accumulation of compatible solutes including proline, glycine betaine, and trehalose. These are not only known to act via osmotic adjustment mechanisms to help maintain cell turgor but are also thought to be able to counteract the harmful effects of reactive oxygen species (ROS) (Ashraf and Foolad, 2007).

*Sorghum bicolor* is an important C_4_ grain crop that is grown in the arid and semi-arid tropics and is known to be particularly well adapted to conditions of low water availability. It is the fifth most important cereal crop grown worldwide, based on yield, and is an important source of food, feed, fibre, and fuel (Kholova *et al.*, 2013). Whilst numerous studies have investigated the physiological mechanisms underlying drought tolerance in sorghum, relatively little is known about the adaptations at the biochemical and molecular level. In the field, agricultural traits conferring drought tolerance have been identified, including the stay-green trait (Rosenow *et al.*, 1983; Sanchez *et al.*, 2002; Thomas and Ougham, 2014). Plants possessing the stay-green trait are able to maintain green photosynthetic leaf area for longer under drought stress conditions at the post-flowering stage and, as a result, produce higher grain yields than their drought-sensitive counterparts (Rosenow *et al.*, 1983; Borrell *et al.*, 2000; Harris *et al.*, 2007). Several sorghum genotypes have been identified that exhibit the stay-green trait, including B35, SC56, and E36-1 (Rosenow *et al.*, 1983; Kebede *et al.*, 2001; Haussmann *et al.*, 2002; Sanchez *et al.*, 2002). Of these, B35 is the best characterized, with a number of physiological studies being carried out on this variety or its derivatives (Crasta *et al.*, 1999; Xu *et al.*, 2000*a*; Kassahun *et al.*, 2010; Vadez *et al.*, 2011).

Previous studies investigating the stay-green trait in sorghum have identified differences in chlorophyll content, transpiration, relative water content (RWC), and nitrogen status when comparing stay-green and senescent lines (Borrell and Hammer, 2000; Thomas and Howarth, 2000; Xu *et al.*, 2000*a*; Harris *et al.*, 2007; Vadez *et al.*, 2011). Other studies have identified differences in tillering and leaf size which could impact upon pre-flowering water usage (Borrell *et al.*, 2014*a*, *b*). As a result, it is thought that the increase in grain yield in the stay-green varieties following stress at the post-flowering stage can be attributed to the emergent consequence of genes acting at the pre-flowering stage (Borrell *et al.*, 2014*a*). Mapping studies based on a number of crosses, largely based on the B35 stay-green line, have also been able to identify four quantitative trait loci (QTLs; Stg1–Stg4) for the trait that are consistent across different backgrounds (Crasta *et al.*, 1999; Subudhi *et al.*, 2000; Xu *et al.*, 2000*b*; Sanchez *et al.*, 2002; Harris *et al.*, 2007). These have been introgressed into the high yielding but senescent R16 background (Kassahun *et al.*, 2010; Vadez *et al.*, 2011). This trait is, however, undoubtedly complex and, despite these advancements, the physiological and molecular basis of this trait remains unclear. Such an understanding would be greatly beneficial not only to improve our understanding of drought tolerance mechanisms in sorghum but also to facilitate the improvement of future sorghum cultivars by marker-assisted selection (MAS).

Transcriptomic analyses, including microarrays, are a valuable way in which mechanistic insights into biological phenomena can be obtained. For example, the comparison of gene expression in different samples can provide insight into the actual biological processes that are perturbed following a specific treatment or between different genotypes. In sorghum, a number of transcriptomic experiments have been carried out recently and have led to the identification of many stress-related transcripts (Zhu-Salzman *et al.*, 2004; Buchanan *et al.*, 2005; Salzman *et al.*, 2005; Park *et al.*, 2006; Dugas *et al.*, 2011; Johnson *et al.*, 2014). The recent release of the sorghum genome sequence (Paterson *et al.*, 2009) and the development of a metabolic pathways database, SorghumCyc (http://pathway.gramene.org/gramene/sorghumcyc.shtml) have greatly facilitated these studies. Microarray analysis could therefore be a powerful approach for elucidating some of the molecular and biochemical pathways involved in conferring the stay-green trait in sorghum.

Here, using microarray analysis, gene expression differences between a stay-green and senescent line are described. Ontological analysis of the differentially expressed genes suggested a potential role for a number of processes in the stay-green trait and in particular a role for proline biosynthesis. This was validated biochemically, and a putative mechanism to explain the higher proline levels in the B35 stay-green line is presented. The data suggest that at least part of the stay-green phenotype of the B35 line is due to increased free proline levels.

## Materials and methods

### Plant growth conditions and sampling of tissue

Seeds of sorghum (*Sorghum bicolor* L. Moench.) R16 and B35 (BT×642) varieties were soaked in water overnight and surface sown singly onto rehydrated 44mm Jiffy peat pellets (LBS Horticulture Ltd, Lancashire, UK). Seedlings were grown in a glasshouse at 28 °C day, 23 °C night, 12h photoperiod, and ~1000 μmol m^–2^ s^–1^. At 30 days after sowing (DAS), the seedlings were transferred to 8 inch pots containing New Horizon Organic and Peat Free Compost (William Sinclair Horticulture Ltd, Lincolnshire, UK). From this point, the photosynthetic efficiency of leaves 2 and 4 was monitored using a portable photosystem efficiency analyser (PEA) machine (Hansatech, Norfolk, UK). A sample from leaf 10 was taken when the average photosynthetic efficiency of leaves 2 and 4 first started to differ between the B35 and R16 varieties, as indicated by a reduced ratio of variable fluorescence (*F*_v_) to maximal fluorescence (*F*_m_) in R16. This occurred at ~45 DAS (Supplementary Fig. S1 available at *JXB* online). At this stage the plants were at the booting stage and had 10 leaves. Leaf 10 was sampled and the tissue was pooled from six plants of each variety. Plants were maintained under well-watered conditions throughout. Experiments were carried out in triplicate, with samples taken on different occasions, to give three biological replicates. Samples were taken at the same time of day for each biological replicate to reduce variation due to circadian/diurnal factors. Tissues samples were harvested into liquid nitrogen and stored at –80 °C.

### Microarray design, and cRNA synthesis and labelling

Total RNA was isolated using the Qiagen miRNeasy Mini Kit with QIAshredder columns for tissue homogenization (Qiagen, Sussex, UK). The integrity of the RNA was confirmed using an Agilent 2100 bioanalyser (Palo Alto, CA, USA) and the RNA 6000 Nano Kit (Agilent). Custom expression microarrays (4×44K format) for sorghum were designed and submitted for manufacturing using the Agilent Technologies eArray web-based application (https://earray.chem.agilent.com/earray/), as described previously (Johnson *et al.*, 2014). All products were obtained from Agilent Technologies UK Ltd (Wokingham, Berkshire, UK) and used according to the Agilent ‘One Colour Low Input Quick Amp Microarray Based gene expression’ protocol, as described previously (Johnson *et al.*, 2014). The labelled cRNA was purified using the RNeasy Mini Kit (Qiagen) according to the manufacturer’s protocol and quantified using a UV-VIS spectrophotometer. The Agilent Hybridization Kit (Cat. no. 5188–5242) was used according to the manufacturer’s instructions, again as described previously (Johnson *et al.*, 2014). The accession number for this microarray is GPL17335.

### Bioinformatic analysis

The Agilent Feature Extraction Software (v10.7) was used to extract data from scanned microarray images. The extracted data were analysed using GeneSpring GX 11 software (Agilent Technologies). Controls, spots of poor quality (not detected), and gene probes which were not present in all three repetitions in either the control or treatment samples were excluded from the analysis. This yielded ~21 000 probes for the B35 versus R16 comparison. From these 21 000 genes, those with an average fold change (FC) of ≥2.0 and a *P*-value of ≤0.05 (moderated *t*-test with Benjamini–Hochberg correction) were selected for further analysis. Singular enrichment snalysis (SEA) of Gene Ontology (GO) terms was determined using AgriGO (http://bioinfo.cau.edu.cn/agriGO/) (Du *et al.*, 2010). Hierarchical clustering of normalized gene expression was carried out on conditions and entities using GeneSpring default settings. The SorghumCyc metabolic pathways database (http://pathway.gramene.org/gramene/sorghumcyc.shtml) was used to identify sorghum genes involved in particular biosynthetic pathways.

### Real-time qPCR

Quantitative PCR (qPCR) validation of the microarray data was carried out using Fluidigm 96 Dynamic arrays (Fluidigm, San Francisco, CA, USA). Three additional biological replicates were set up and sampled as described above. Assays were run in triplicate to give three technical replicates of each biological replicate. The set-up was performed in accordance with the ‘Fluidigm^®^ 96.96 Real-Time PCR Workflow’ (PN68000088) (http://fgl.salk.edu/BioMark/pdf/96.96%20Real-Time%20PCR%20Workflow%20Quick%20Reference%20rev%20C1.pdf). Total RNA (1 μg) was used as input in a 20 μl reverse transcription reaction. The SuperScript III First-Strand Synthesis SuperMix Kit (Applied Biosystems, Foster City, CA, USA, Cat. no. 11752-050) was used for first-strand cDNA synthesis, and the TaqMan PreAmp Master Mix (Applied Biosystems) was used for pre-amplification of the cDNA. Custom-designed 20×Custom TaqMan^®^ Gene Expression Assays (Applied Biosystems) were used for amplification of the cDNA, and data were collected using Fluidigm Real-Time PCR analysis Software v3.0.2 (see Supplementary Table S1 at *JXB* online for a full list of probes). Relative quantification was accomplished using the comparative Ct method (ΔΔC_T_ method) (Livak and Schmittgen, 2001). Sb04g028990.1 was used as an endogenous control due to its unchanging expression following various stress treatments in previous microarray analyses (Johnson *et al.*, 2014). qPCR of the *P5CS* transcripts was carried out using an AB 7300 real-time PCR system (Applied Biosystems) and GoTaq qPCR master mix (Promega, Madison, WI, USA) as described previously (Moffat *et al.*, 2012). The oligo sequences used (sequence direction 5′ to 3′) were: Sb03g039820.1 Fwd, TCACCAGATGAACGCAAA; Sb03g039820.1 Rev, CCTCAACATCGCTTCATTT; Sb09g022290.1 Fwd, GCGTCTTTAGCAATCC GAAG; Sb09g022290.1 Rev, AAGTTTT TCACCCACGT TGC; Sb09g022310.1 Fwd, ATTCAGCTCCATC ACCTGCT; and Sb09g022310.1 Rev, CATCATCAAGTTG GGCACTG.

### Osmotic stress and proline quantification

Osmotic stress was administered at 14 DAS by application of 10% polyethylene glycol (PEG) to peat plugs, as described previously (Turkan *et al.*, 2005). Leaf samples were taken following 3 d of water withdrawal and used both for RNA extraction for qPCR and for proline quantification. Proline levels were quantified using ultra-high perfomance liquid chromatography [UPLC; Waters Acquity H-Class UPLC^**®**^ system with fluorescence (FLR) and photodiode array (PDA) detectors; Waters, Wilmslow, UK]. Leaf tissue was ground in liquid nitrogen and lyophilized overnight. Lyophilized tissue (0.04g) was extracted in 1.5ml of 0.1 N HCl by grinding and then centrifugation at 17 000 *g* for 20min at 4 °C. The extracts were then sequentially derivatized with OPA (*o*-phthaldialdehyde) reagent and FMOC (fluorenylmethyloxycarbonyl) (Sigma). OPA reagent consists of 260mM *N*-isobutyryl-l-cysteine (IBLC) (Sigma) and 170mM OPA (Sigma) in 1M potassium borate buffer (pH 10.4). The following reactions were set up in an HPLC vial: 10 μl of sample, 10 μl of OPA reagent, 20 μl of FMOC (5mM in acetonitrile), and 60 μl of 100% methanol. Separations were performed on a Cortecs C18, 100 mm×2.1mm, 1.6 μm column (Waters), and elution was achieved at 40 °C. Mobile phase A was made up of 20mM sodium acetate, pH 6.0. Mobile phase B was made up of acetonitrile:methanol:water in a 45:45:10 (v/v/v) ratio. A flow rate of 400 μl min^–1^ was used. Automated HPLC injection added 3 μl of the sample for analysis, and samples were run for 20min. For OPA detection, the excitation and emission wavelengths were 340nm and 455nm, respectively. For FMOC detection, the excitation and emission wavelengths were 266nm and 305nm, respectively. Quantification was made with reference to a proline standard that was derivatized and run with each sample set.

### Stay-green QTLs and SNP identification

Genes within known QTLs for stay-green were identified using the Comparative Saccharinae Genome Resource (CSGR) (http://helos.pgml.uga.edu/qtl/) (Zhang *et al.*, 2013). In order to compare the upstream promoter sequence, gDNA was extracted from the R16 and B35 varieties using the Dellaporta method (Dellaporta *et al.*, 1983). PCR was carried out using BioTaq Polymerase (BIO-21040) and the following primers (sequence direction 5′ to 3′): –500Fwd, TTGTGGTCGTGTGGCACGT; –500 Rev, CGGGGGGGAATACTGGTGGGATC. Sequencing was performed by the Durham University sequencing service, and sequences were aligned using ClustalW2 (www.ebi.ac.uk/Tools/msa/clustalw2/).

## Results

### Microarray analysis identified genes differentially expressed in a stay-green (B35) versus a senescent line (R16) at the pre-flowering stage

In an attempt to better understand the molecular processes that are associated with the stay-green trait, microarray analysis was carried out to capture gene expression differences between a stay-green and senescent line. Tissue samples were taken at ~45 DAS under well-watered conditions. At this time point there were higher levels of chlorophyll in B35 compared with R16, as indicated by measurements of chlorophyll fluorescence (see Supplementary Fig. S1 at *JXB* online); therefore, at least one known element of the trait was manifesting at this time point (Thomas and Howarth, 2000). Samples were harvested on three separate occasions to provide three biological replicates.

**Fig. 1.**
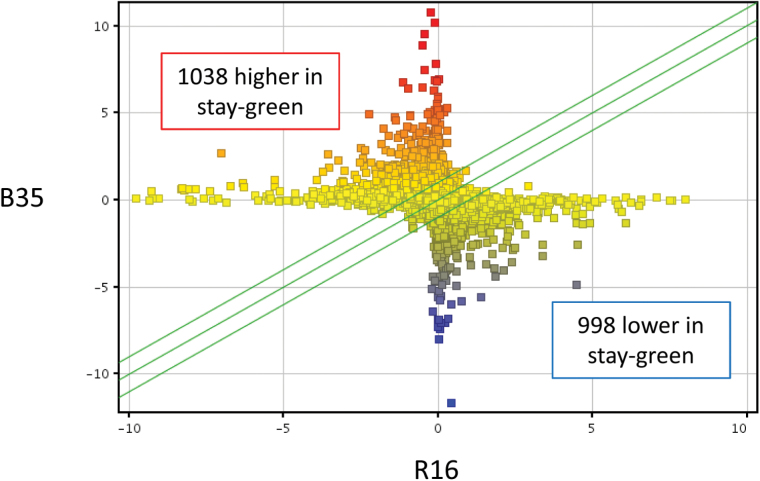
Scatter plots showing the distribution of expression of filtered genes in the stay-green (B35) line compared with the senescent (R16) line. Axes denote normalized gene expression and squares represent individual genes. The green lines mark a 2-fold cut-off value. Differentially expressed genes are based on a 2-fold cut-off and a *P*-value <0.05. Colour corresponds to normalized gene expression, with red representing high relative expression and blue representing low relative gene expression.

**Fig. 2.**
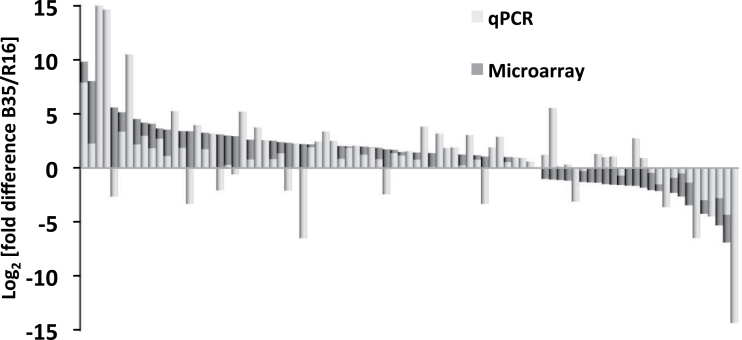
Comparison of fold changes obtained by microarray analysis and by qPCR. Bars represent the Log_2_ of the fold change when comparing expression in the stay-green (B35) and the senescent (R16) varieties.

RNA was hybridized to custom-designed microarray chips containing 28 585 gene probes, as used previously (Johnson *et al.*, 2014). As shown in Fig. 1, 1038 genes were expressed to higher levels (FC>2, *P*<0.05) in B35 compared with R16, and 998 genes were expressed to lower levels (Supplementary Tables S2, S3 at *JXB* online). These gene expression changes constitute 3.6% and 3.4% of total genes on the chip for the genes expressed to higher and lower levels, respectively. Differentially expressed genes identified in the microarray were validated using qPCR on an additional three biological replicates. Probes were designed to 87 genes and, of these, 83% showed a fold change in the same direction in both the qPCR and microarray analysis (see Fig. 2; Supplementary Table S4). The qPCR largely confirmed the results obtained by the microarray analysis, confirming the accuracy of the high-density microarrays and the robustness of the experimental system.

### Ontological analysis of differentially expressed genes identified enriched biological processes in the stay-green B35 line

**Fig. 3.**
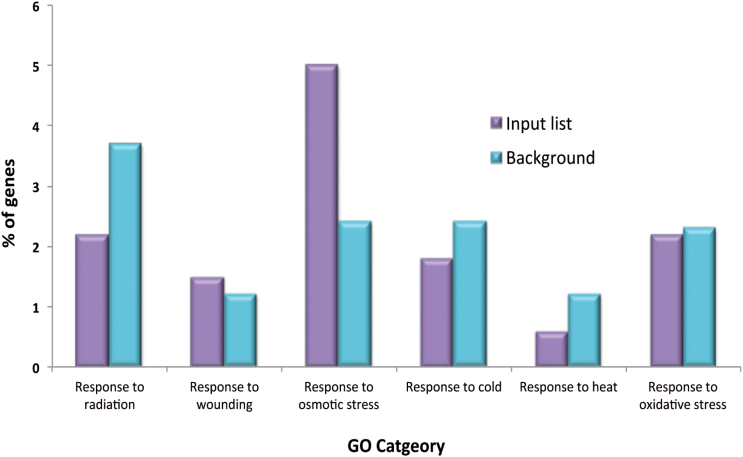
Singular enrichment analysis of genes expressed at higher levels (FC>2, *P*<0.05) in the stay-green (B35) variety compared with the senescent (R16) variety. The bar chart shows the percentage of the genes expressed to higher levels in B35 that are associated with different stress stimuli (Input list) compared with the percentage of genes in the genome involved with that same stimulus (Background).

In order to identify the biological processes and molecular functions that are enriched within the differentially expressed gene sets, GO analysis was carried out. The AgriGO gene ontology tool (http://bioinfo.cau.edu.cn/agriGO/) was used to group genes into broad functional categories based on their GO annotations (Du *et al.*, 2010). Singular enrichment analysis (SEA) was then carried out to identify particular GO categories that were significantly enriched (*P*<0.05) within the genes expressed at higher levels in B35 (Table 1). Enriched GO categories include processes such as ‘post-embryonic morphogenesis’ and ‘anatomical structure homeostasis’. Other enriched GO categories include ‘cell redox homeostasis’ and ‘cellular amino acid metabolic activity’. Notably, processes that are known to be associated with the plant response to low water availability were also enriched, including ‘response to osmotic stress’ and ‘water transport’. In order to determine whether genes associated with other known stress stimuli were differentially expressed, the percentage of genes associated with these stimuli was compared with the percentage of genes in the genome that are associated with that same stimulus. The ‘response to osmotic stress’ category was found to be the only strongly enriched stimulus, with nearly 5% of the genes in the input list belonging to this category. This is particularly interesting given that sorghum lines that have the stay-green trait are better able to survive under conditions of low water availability. The response to wounding category showed slight enrichment; however, there was no enrichment of genes associated with any of the other stimuli (Fig. 3).

**Table 1.** GO analysis of genes expressed at higher levels in B35 versus R16 (>2 fold; P-value<0.05)

GO annotation	GO term	Count in selection (out of 1038)	Count in total genome (out of 26 245)	Fold enrichment	*P*-value	FDR
Biological process						
GO:0010876	Lipid localization	7	16	11.06	3.00E-07	0.00033
GO:0015692	Lead ion transport	9	33	6.90	6.70E-07	0.00055
GO:0009886	Post-embryonic morphogenesis	16	122	3.32	2.00E-06	0.0013
GO:0048448	Stamen morphogenesis	9	38	5.99	2.50E-06	0.0013
GO:0010037	Response to carbon dioxide	7	22	8.04	3.80E-06	0.0018
GO:0060249	Anatomical structure homeostasis	5	10	12.64	7.30E-06	0.003
GO:0009719	Response to endogenous stimulus	92	1755	1.33	8.70E-06	0.0032
GO:0006970	Response to osmotic stress	42	631	1.68	1.20E-05	0.0038
GO:0048466	Androecium development	15	126	3.01	1.40E-05	0.0038
GO:0048443	Stamen development	15	126	3.01	1.40E-05	0.0038
GO:0048449	Floral organ formation	11	72	3.86	1.80E-05	0.0045
GO:0006026	Aminoglycan catabolic process	8	39	5.19	2.80E-05	0.0061
GO:0006032	Chitin catabolic process	8	39	5.19	2.80E-05	0.0061
GO:0006833	Water transport	5	13	9.72	3.40E-05	0.0067
GO:0010033	Response to organic substance	104	2117	1.24	3.40E-05	0.0067
GO:0006030	Chitin metabolic process	8	42	4.82	4.90E-05	0.0078
GO:0042044	Fluid transport	5	14	9.03	5.20E-05	0.0078
GO:0009725	Response to hormone stimulus	82	1601	1.30	5.00E-05	0.0078
GO:0006855	Multidrug transport	13	109	3.02	4.90E-05	0.0078
GO:0015893	Drug transport	14	125	2.83	5.20E-05	0.0078
GO:0042493	Response to drug	17	185	2.32	0.00011	0.016
GO:0042398	Cellular amino acid derivative biosynthetic process	31	460	1.70	0.00012	0.016
GO:0042221	Response to chemical stimulus	145	3244	1.13	0.00013	0.017
GO:0006022	Aminoglycan metabolic process	9	61	3.73	0.00013	0.017
GO:0048444	Floral organ morphogenesis	11	90	3.09	0.00015	0.018
GO:0045454	Cell redox homeostasis	15	156	2.43	0.00016	0.019
GO:0009607	Response to biotic stimulus	69	1355	1.29	0.0002	0.021
GO:0048465	Corolla development	9	65	3.50	0.00022	0.021
GO:0051554	Flavonol metabolic process	6	28	5.42	0.00022	0.021
GO:0051555	Flavonol biosynthetic process	6	28	5.42	0.00022	0.021
GO:0051552	Flavone metabolic process	6	28	5.42	0.00022	0.021
GO:0051553	Flavone biosynthetic process	6	28	5.42	0.00022	0.021
GO:0048441	Petal development	9	65	3.50	0.00022	0.021
GO:0042592	Homeostatic process	36	589	1.55	0.00024	0.021
GO:0010149	Senescence	13	127	2.59	0.00023	0.021
GO:0006575	Cellular amino acid derivative metabolic process	38	645	1.49	0.00034	0.029
GO:0010260	Organ senescence	12	119	2.55	0.00046	0.039
Molecular function						
GO:0030613	Oxidoreductase activity, acting on phosphorus or arsenic in donors	9	29	7.85	2.00E-07	5.50E-05
GO:0008794	Arsenate reductase (glutaredoxin) activity	9	29	7.85	2.00E-07	5.50E-05
GO:0016491	Oxidoreductase activity	127	2349	1.37	6.30E-08	5.50E-05
GO:0030614	Oxidoreductase activity, acting on phosphorus or arsenic in donors, with disulphide as acceptor	9	29	7.85	2.00E-07	5.50E-05
GO:0030611	Arsenate reductase activity	9	33	6.90	6.70E-07	0.00015
GO:0008061	Chitin binding	7	19	9.32	1.20E-06	0.0002
GO:0016758	Transferase activity, transferring hexosyl groups	43	595	1.83	1.30E-06	0.0002
GO:0016757	Transferase activity, transferring glycosyl groups	49	768	1.61	7.80E-06	0.00097
GO:0008194	UDP-glycosyltransferase activity	28	341	2.08	7.70E-06	0.00097
GO:0005544	Calcium-dependent phospholipid binding	6	17	8.92	9.70E-06	0.001
GO:0004553	Hydrolase activity, hydrolysing *O*-glycosyl compounds	38	543	1.77	9.90E-06	0.001
GO:0008171	*O*-Methyltransferase activity	11	71	3.92	1.60E-05	0.0015
GO:0004568	Chitinase activity	7	32	5.53	5.70E-05	0.0042
GO:0030247	Polysaccharide binding	7	32	5.53	5.70E-05	0.0042
GO:0001871	Pattern binding	7	32	5.53	5.70E-05	0.0042
GO:0016798	Hydrolase activity, acting on glycosyl bonds	38	595	1.61	6.90E-05	0.0048
GO:0015035	Protein disulphide oxidoreductase activity	10	71	3.56	8.70E-05	0.0057
GO:0009055	Electron carrier activity	54	984	1.39	0.00015	0.0095
GO:0015036	Disulphide oxidoreductase activity	10	84	3.01	0.00036	0.021
GO:0016656	Monodehydroascorbate reductase (NADH) activity	5	22	5.75	0.00055	0.031
GO:0008146	Sulphotransferase activity	5	23	5.50	0.00069	0.037
GO:0005506	Iron ion binding	47	890	1.34	0.00083	0.042
GO:0005543	Phospholipid binding	10	95	2.66	0.00095	0.046
Cellular component						
GO:0005576	Extracellular region	46	763	1.52	5.40E-05	0.029

**Fig. 4.**
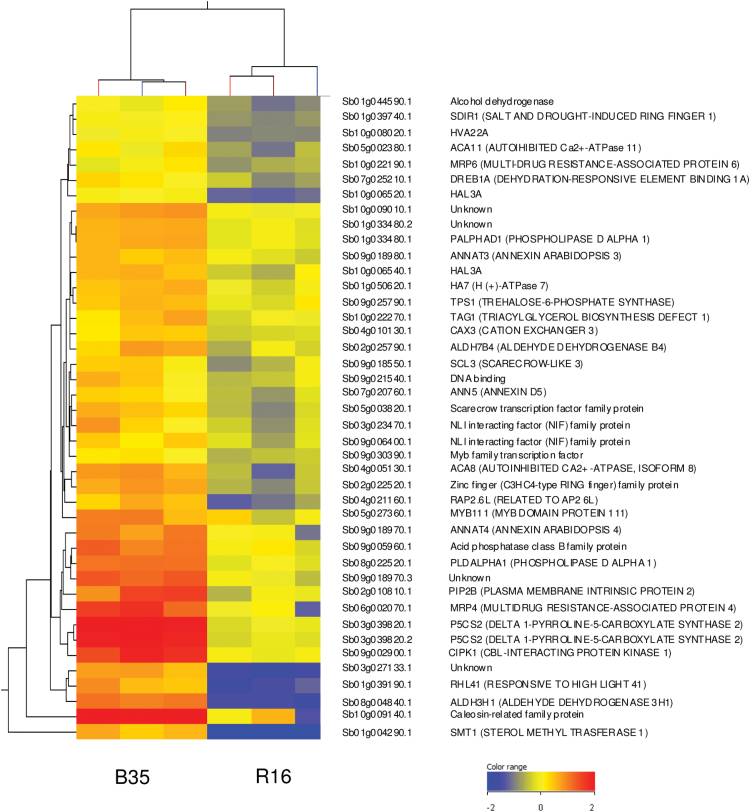
Heat map showing the genes expressed at higher levels in the stay-green (B35) variety compared with the senescent (R16) variety that are associated with the ‘response to osmotic stress’ GO category. Colour denotes normalized expression values.

The 42 genes that are involved specifically in the plant ‘response to osmotic stress’ were identified and their expression levels analysed (Fig. 4). This list contains genes encoding a dehydration-responsive element-binding (*DREB1A*) transcription factor, a ubiquitin ligase called salt and drought-induced RING finger 1 (*SDIR1*), and a CBL-interacting serine/threonine-protein kinase 1 (*CIPK1*). Other up-regulated genes include those encoding trehalose-6-phosphate synthase (*TPS*) and delta-1-pyrroline-5-carboxylate-synthase (*P5CS2*), which are known to be important for the biosynthesis of trehalose and proline, respectively (Goddijn and van Dun, 1999; Ashraf and Foolad, 2007). Whilst not all of the enriched processes listed in Table 1 will necessarily be causal to the stay-green phenotype, it is possible that the higher expression of genes involved with the plant response to osmotic stress in B35 may be contributing to its ability to maintain green leaf area for longer under drought conditions.

### Genes that are associated with the biosynthesis of proline are expressed at higher levels in the B35 stay-green line compared with a senescent variety

**Fig. 5.**
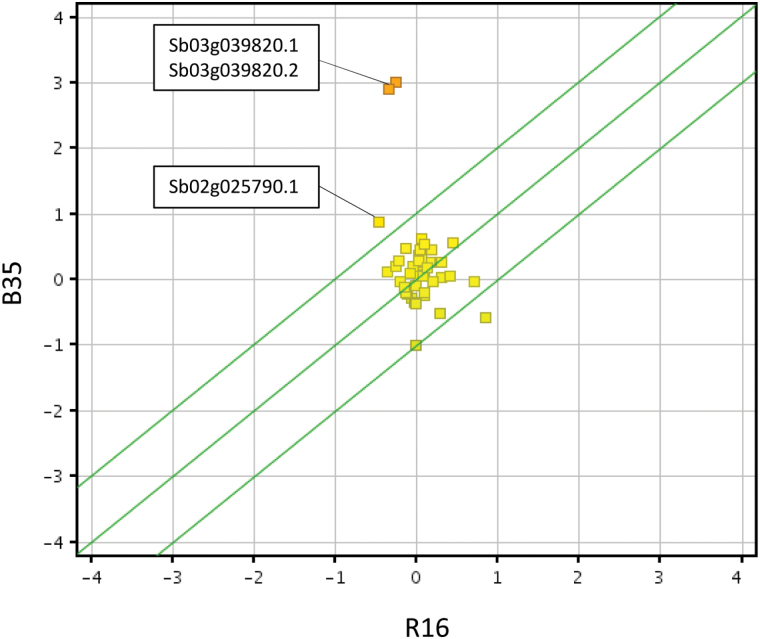
Scatter plots showing the distribution of expression of filtered genes involved in proline biosynthesis. Genes were identified using the SorghumCyc metabolic pathways database. Axes denote normalized gene expression and squares represent individual genes. The green lines mark a 2-fold cut-off value.

Amongst the 42 genes presented in Fig. 4, *P5CS2* had the fifth highest fold change when comparing B35 and R16. The biosynthesis of proline is known to play an important role in the drought stress response (Ashraf and Foolad, 2007). Given the large difference in the expression of this gene (~8.7-fold), the proline biosynthesis pathway was investigated further. The SorghumCyc metabolic pathways database (http://pathway.gramene.org/gramene/sorghumcyc.shtml) was used to identify all sorghum genes involved in the biosynthesis of proline. The expression of these genes was then compared between B35 and R16 (Fig. 5). The expression of three genes associated with the biosynthesis of proline was found to be higher in B35 compared with R16 (Fig. 5). These genes correspond to two different *P5CS2* transcripts (Sb03g039820.1 and Sb03g039820.2) and a glutamate *S*-semialdehyde dehydrogenase (Sb02g025790.1). These same genes were also expressed at higher levels in B35 at a younger seedling stage (Table 2).

**Table 2.** Genes associated with proline biosynthesis that are expressed at higher levels in B35 versus R16

SbID	Gene name	FC (Abs) in B35 versus R16 at 50 DAS	FC (Abs) in B35 versus R16 at 14 DAS
Sb03g039820.1	Delta1-pyrroline-5-carboxylase-synthestase (*P5CS2*)	8.74	2.85
Sb03g039820.2	Delta1-pyrroline-5-carboxylase-synthestase (*P5CS2*)	8.55	2.52
Sb02g025790.1	Glutamate *S*-semialdehyde dehydrogenase	2.32	3.35

**Fig. 6.**
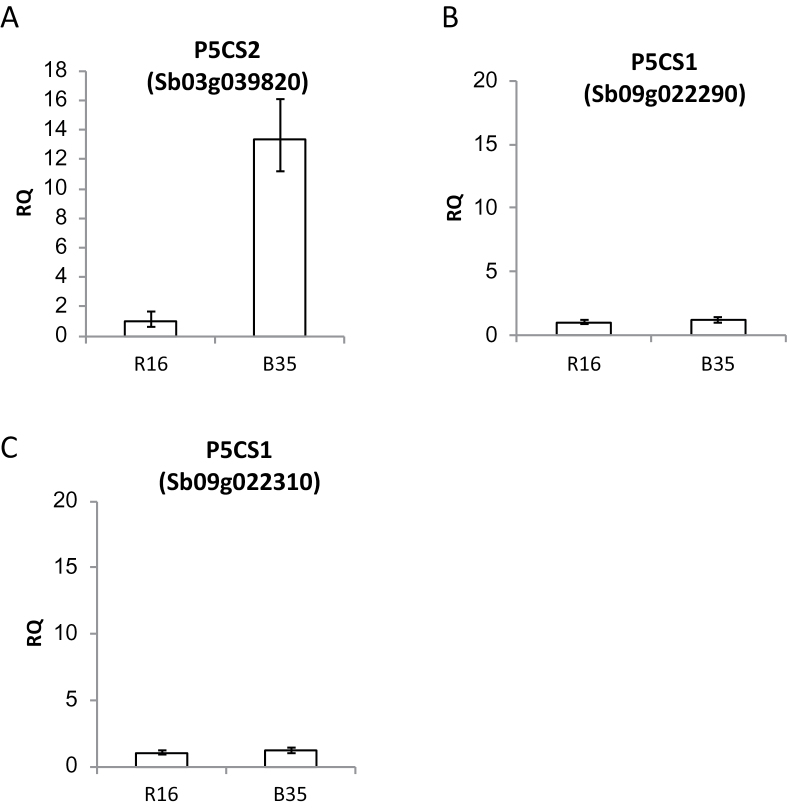
Relative transcript abundance of (A) Sb03g039820.1, (B) Sb09g022290.1, and (C) Sb09g022310.1 in the senescent (R16) and stay-green (B35) varieties at 45 DAS. Each of these genes has been annotated as P5CS in the sorghum genome. Error bars represent RQ_MIN_ and RQ_MAX_, and constitute the acceptable error level for a 95% confidence level according to Student’s *t*-test.

The *P5CS2* gene encodes an enzyme responsible for the rate-limiting step in proline biosynthesis (Kishor *et al.*, 1995). Three *P5CS* genes have been identified in the sorghum genome. In order to confirm that only one of these genes was expressed to higher levels in B35, qPCR, with probes designed specifically for each gene, was carried out and gene expression was compared in B35 and R16. Of the three annotated *P5CS* genes predicted in the sorghum genome, only *P5CS2* (Sb03g039820.1) was expressed at higher levels in the B35 line (Fig. 6).

### The B35 stay-green line has higher proline levels than the R16 senescent line under both well-watered and osmotically stressed conditions

**Fig. 7.**
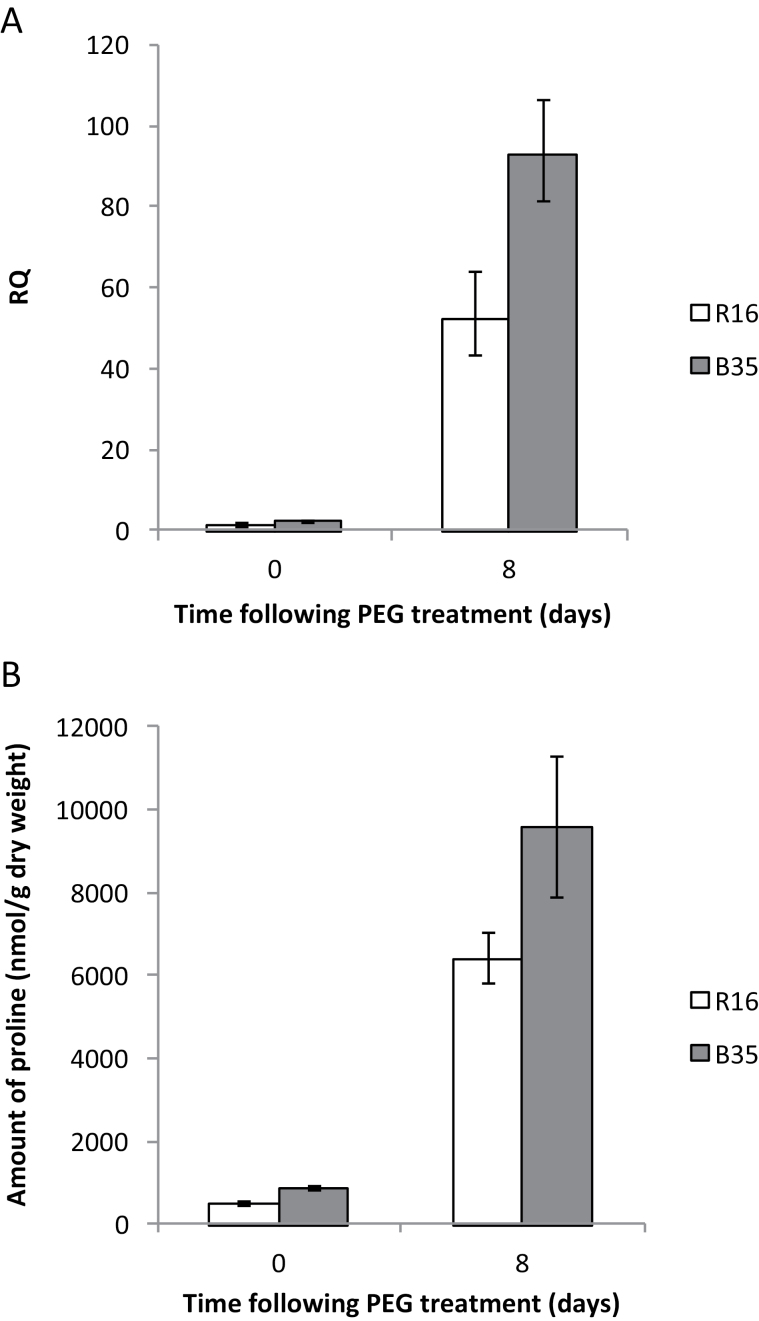
(A) Representative graph showing relative transcript abundance of *P5CS2* in the stay-green (B35) and senescent (R16) lines following PEG treatment. Error bars represent RQ_MIN_ and RQ_MAX_, and constitute the acceptable error level for a 95% confidence level according to Student’s *t*-test. (B) Amount of proline in the stay-green (B35) and senescent (R16) sorghum varieties following PEG treatment at 14 DAS. Error bars show the SE (*n*=5).

Differences in gene expression do not always correlate with changes at the protein or metabolite level. Therefore, in order to determine whether the observed differences in *P5CS2* gene expression result in an increase in actual proline levels, total proline content was quantified in B35 and R16 under both well-watered and osmotically stressed conditions using HPLC. Proline levels were found to be ~1.8-fold higher in the B35 stay-green line compared with the R16 senescent line under well-watered conditions and ~1.5-fold higher under osmotically stressed conditions (Fig. 7). The differences in *P5CS* gene expression therefore correlate well with the actual proline levels.

### *P5CS2* is found within a known QTL for the stay-green trait and contains an SNP in the promoter region

Genes within known QTLs for the stay-green trait were identified using the CSGR (http://helos.pgml.uga.edu/qtl/). The majority of the stay-green QTLs in this database were identified using B35 as the source of stay-green (Tuinstra *et al.*, 1997; Crasta *et al.*, 1999; Subudhi *et al.*, 2000; Xu *et al.*, 2000*b*; Sanchez *et al.*, 2002; Harris *et al.*, 2007). This list of genes was compared with the list of differentially expressed genes identified in the microarrays. Out of the 2036 differentially expressed genes identified in the arrays, 289 are within a known QTL for stay-green (see Supplementary Tables S5 and S6 at *JXB* online for a full list). Interestingly for this study, *P5CS2* also lies within a stay-green QTL region that is now known as Stg1 (Subudhi *et al.*, 2000; Xu *et al.*, 2000*b*).

**Fig. 8.**
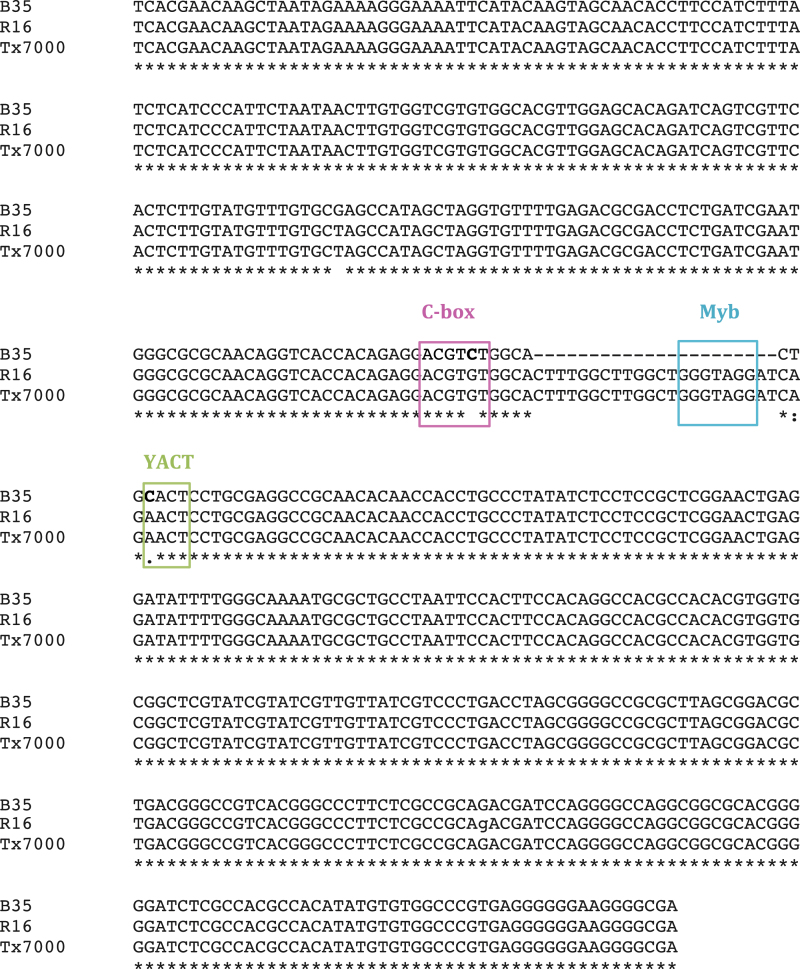
Sequence alignment of base pairs –550 to –13 upstream of P5CS2 in the stay-green (B35) and senescent varieties (R16 and Tx7000). (This figure is available in colour at *JXB* online.)

Differences in the expression of *P5CS2* between the varieties could be associated with polymorphisms in the upstream promoter region. To test this, 500bp upstream of the start codon was amplified using PCR and then sequenced. The sequences from the stay-green B35 variety and two senescent varieties, R16 and Tx7000, were then compared (Fig. 8). Sequence alignment identified three single nucleotide polymorphisms (SNPs) and a 22bp deletion within the B35 line when compared with both senescent varieties (Fig. 8). Two of the identified SNPs lie within known *cis*-element motifs. For example, a G to C SNP can be found in a potential C-box motif (Simpson *et al.*, 2003) and an A to C SNP in a YACT motif (Gowik *et al.*, 2004). A potential Myb element within the B35 upstream sequence is not present in the senescent R16 and Tx7000 varieties (Grotewold *et al.*, 1994). It is therefore possible that differences in the promoter sequence of *P5CS2* in B35 may be responsible for the differences in the expression of this gene.

## Discussion

The identification of genes conferring drought tolerance and in particular those underlying specific traits for drought tolerance will be very important in the future in order to reduce the adverse impacts of drought stress on crop yields. In previous studies, transcriptomic analysis has proven to be a powerful approach for the identification of stress-induced genes in sorghum (Buchanan *et al.*, 2005; Salzman *et al.*, 2005; Dugas *et al.*, 2011; Johnson *et al.*, 2014). Here, microarray analysis was used to compare gene expression between a drought-tolerant stay-green sorghum line and a drought-sensitive senescent line in order to identify genes and biological processes that are putatively associated with the important stay-green trait. Gene expression was compared under well-watered conditions because many previously observed differences in physiology between stay-green and senescent lines were identified under non-stressed conditions (i.e. differences in chlorophyll content and transpiration efficiency) (Kassahun *et al.*, 2010; Vadez *et al.*, 2011; Borrell *et al.*, 2014*a*).

A total of 1038 genes were found that are expressed at higher levels in the stay-green line and 998 genes that are expressed at lower levels (Fig. 1). Whilst not all of these will necessarily be causal to the stay-green phenotype, an analysis of these genes and the processes they are involved with provides a valuable insight into the mechanisms underlying the trait. Ontological analysis of these genes identified enriched biological processes, including ‘response to osmotic stress’ and ‘water transport’ (Table 1). Stay-green sorghum lines are known to be able to survive for longer under conditions of low water availability (Rosenow *et al.*, 1983; Xu *et al.*, 2000*a*). For this reason, genes that are known to provide protection against these conditions, such as those in the ‘response to osmotic stress’ category, provide obvious initial candidates as contributors to the phenotype.

An interesting gene found within the ‘response to osmotic stress’ GO category was *P5CS2*. The products of *P5CS* genes are known to be involved in the conversion of glutamate to pyyroline-5-carboxylate and as such they act as the rate-limiting step in the biosynthesis of proline (Kishor *et al.*, 1995). Not only was the expression of this gene shown to be higher in the stay-green line but this higher expression resulted in constitutively higher levels of actual proline levels (Fig. 7). Proline is known to accumulate in plants, including sorghum, under low water conditions, and is known to have a number of protective properties including a role in osmotic adjustment, detoxification of ROS, protection of membrane integrity, and stabilization of proteins (Wood *et al.*, 1996; Hsu *et al.*, 2003; Ashraf and Foolad, 2007; Su *et al.*, 2011; Damame *et al.*, 2014). In addition, evidence has suggested that proline is able to induce the expression of stress-responsive genes which possess proline-responsive elements (PREs) in their promoters (Satoh *et al.*, 2002). As a consequence, the overexpression of *P5CS* genes and the accumulation of proline have been shown to result in drought tolerance in a wide range of species (Kishor *et al.*, 1995; Hong *et al.*, 2000). In addition, proline concentrations have also been shown to be generally higher in stress-tolerant genotypes of plants, including sorghum (Sivaramakrishnan, 1988; Hsu *et al.*, 2003; Nayyar and Walia, 2003; Ashraf and Foolad, 2007; Damame *et al.*, 2014). The proline accumulation identified here could therefore be an important way in which stay-green lines are able to withstand drought stress for longer and therefore maintain their green leaf area.

The maintenance of cell turgor via osmotic adjustment is particularly important during cell growth and leaf expansion. Sorghum plants with a better capacity for osmotic adjustment have been shown to have a larger leaf area and have better leaf retention during grain filling (Tangpremsri *et al.*, 1995). Stay-green sorghum lines have been shown to have higher RWC than senescent lines (Xu *et al.*, 2000*a*). It is possible, therefore, that the high proline accumulation identified here in the stay-green variety is contributing to the maintenance of high RWC. This could help with the production of a strong canopy that is better able to intercept light. This means that when fresh water availability is reduced at later growth stages, the stay-green varieties are better adapted to cope. Some evidence has suggested that stay-green lines have a modified root architecture (Borrell *et al.*, 2014*a*). Whilst not investigated specifically in this study, it is also plausible that if *P5CS2* is additionally expressed at higher levels in the roots, osmotic adjustment could enable better root growth, which could facilitate water uptake. Stay-green sorghum plants are able to save water in the period prior to flowering (Xu *et al.*, 2000*a*). The accumulation of compatible solutes such as proline could again be associated with conferring these phenotypes.

Given the complexity of the stay-green trait, however, it is perhaps unlikely that only one gene or process is involved in conferring the trait. Indeed, at least four QTLs have been identified, and individual introgressions of these QTLs all show the stay-green phenotype, albeit to different extents (Subudhi *et al.*, 2000; Sanchez *et al.*, 2002; Kassahun *et al.*, 2010; Vadez *et al.*, 2011). There are therefore at least four different contributing genetic regions. Studies by Borrell *et al.* suggest that developmental differences could be a contributing factor. For example, stay-green near-isogenic lines (NILs) have been shown to display reduced tillering and reduced size of upper leaves when compared with their recurrent parent Tx7000 (Borrell *et al.*, 2014*a*). These differences are thought to reduce crop water usage prior to anthesis, resulting in greater water availability at the post-flowering stage. Interestingly, the present results show an enrichment of the ‘anatomical structure homeostasis’ and ‘post-embryonic morphogenesis’ GO categories amongst the highly expressed genes. Within these categories are genes associated with auxin biosynthesis and transport (see Supplementary Table S2 at *JXB* online). The plant hormone auxin is known to play a role in shoot branching and leaf development (Dengler and Kang, 2001; McSteen and Leyser, 2005); therefore, it is possible that these identified auxin-related gene expression differences may be contributing to the developmental differences previously described.

Multiple genes may be acting co-operatively to bring about the stay-green phenotype. Other interesting genes identified in this study include the signalling gene *SDIR1*. Homologues of this *SDIR1* gene have been shown in *Arabidopsis*, maize, and rice to confer drought tolerance when overexpressed by influencing stomatal aperture and water loss by transpiration (Zhang *et al.*, 2007, 2008; Xia *et al.*, 2012). Given the known differences in transpiration efficiency and pre-anthesis water usage between stay-green and senescent lines (Xu *et al.*, 2000*a*; Vadez *et al.*, 2011), it is possible that higher levels of SDIR1 are resulting in reduced transpiration and could therefore be contributing to this aspect of the stay-green phenotype. The *DREB1A* transcription factor gene was also expressed at higher levels in the stay-green line. Transcription factors act as master regulators and can influence the expression of numerous downstream genes. DREB transcription factors have been shown in a number of species to influence stress-related gene expression, and their overexpression has been shown to increase stress tolerance in wheat and rice (Ito *et al.*, 2003; Shen *et al.*, 2003).

Numerous studies have led to the identification of QTLs for the stay-green trait, and four of these QTLs are known to be consistent across multiple environments (Crasta *et al.*, 1999; Xu *et al.*, 2000*b*; Sanchez *et al.*, 2002). Here, the genes that were differentially expressed between the stay-green and senescent varieties were compared with genes known to be within the Stg QTL regions in the genome. This approach of combining QTL and microarray analysis is a powerful one for the identification of candidate genes for a trait and has contributed significantly to candidate gene identification (Pandit *et al.*, 2010; Yano *et al.*, 2012). Out of the 2036 differentially expressed genes, 286 were found to lie within a stay-green QTL region. Whilst not all of the differentially expressed genes are within the QTL intervals, and so clearly not candidates, many may act downstream of these genes and therefore could act as diagnostic markers for trait selection. Interestingly, the *P5CS2* gene previously described can be found within the Stg1 QTL interval. Sequencing of the putative *P5CS2* promoter in both stay-green and senescent varieties enabled the identification of three SNPs within the B35 promoter along with a 22bp deletion (Fig. 8). These polymorphisms co-locate with known *cis*-element motifs. For example, a G to C SNP was identified within an ABRE-like sequence, ACGTG, which is known to be involved in the induction of genes associated with the response to dehydration stress (Simpson *et al.*, 2003). The A to C SNP also lies within a YACT motif (Gowik *et al.*, 2004). It is possible that these polymorphisms underlie the QTL and are responsible for the differences in the expression of *P5CS2* between the stay-green and senescent varieties.

Further verification of candidate genes in the other QTL regions, by genome sequencing and by the analysis of gene function though the production of transgenics, will be an important next step to validate their importance. It is important to bear in mind here that the gene expression comparison described is between only two genotypes; one stay-green and one senescent. B35 was selected as a source of stay-green for this study due to the fact that the majority of QTL mapping experiments have used this line (Tuinstra *et al.*, 1997; Subudhi *et al.*, 2000; Xu *et al.*, 2000*b*). However, it will be important to investigate gene expression changes in other stay-green sources. In particular, the use of NILs which differ genetically in one or more QTL only will be particularly beneficial for the validation of candidate genes. Some evidence suggests that some Stg QTLs in sorghum overlap with QTLs for nodal root angle, and it has been suggested that this could enhance water uptake in the stay-green lines (Mace *et al.*, 2012; Borrell *et al.*, 2014*a*). It will also therefore be important to investigate the role of the roots and root signalling in conferring the phenotype. The stay-green trait in sorghum is an extremely complex phenotype which is confounded by strong genotype and environment interactions (Vadez *et al.*, 2011); therefore, it will also be important to investigate gene expression changes at different developmental stages, in different growth environments, and in different genetic backgrounds.

In conclusion, these results provide an excellent starting point for the identification of genes underlying this important agricultural trait and could help facilitate breeding for stay-green via MAS or via the production of more targeting introgression lines in the future.

## Supplementary data

Supplementary data are available at *JXB* online

Figure S1. Measurements of *F*_v_/*F*_m_ in R16 and B35 from 35 DAS. Samples were taken for the microarray analysis at ~45 DAS.

Table S1. qPCR probes used for validation of the microarray data.

Table S2. Genes expressed to higher levels (FC>2, *P*<0.05) in B35 versus R16 at 50 DAS.

Table S3. Genes expressed to lower levels (FC>2, *P*<0.05) in B35 versus R16 at 50 DAS.

Table S4. B35 versus R16 gene expression changes obtained using qPCR and microarray analysis.

Table S5. Genes that are expressed to higher levels (FC>2, *P*<0.05) in B35 versus R16 at 50 DAS and that lie within the Stg QTL region

Table S6. Genes that are expressed to lower levels (FC>2, *P*<0.05) in B35 versus R16 at 50 DAS and that lie within the Stg QTL region

Supplementary Data

## Abbreviations:

DAS: days after sowing
FC: fold change
GO: Gene Ontology
MAS: marker-assisted selection
NIL: near-isogenic line
PEA: photosystem efficiency analyser
QTL: quantitative trait locus
ROS: reactive oxygen species
RWC: relative water content
SEA: singular enrichment analysis
SNP: single nucleotide polymorphism.
