# Supplementary material for: Transcriptomic analysis comparing stay-green and senescent Sorghum bicolor lines identifies a role for proline biosynthesis in the stay-green trait
Source: J Exp Bot. 2015 Aug 28;66(22):7061–73. doi: 10.1093/jxb/erv405 (PMC4765785; doi:10.1093/jxb/erv405)
Supplement: Supplementary Data [file supp_66_22_7061__index.html]

Transcriptomic analysis comparing stay-green and senescent Sorghum bicolor lines identifies a role for proline biosynthesis in the stay-green trait — Transcriptomic analysis comparing stay-green and senescent Sorghum bicolor lines identifies a role for proline biosynthesis in the stay-green trait — Supplementary Data 

# Transcriptomic analysis comparing stay-green and senescent *Sorghum bicolor* lines identifies a role for proline biosynthesis in the stay-green trait

## Supplementary Data

Data files

- Supplementary Data - Supplementary Data
- Supplementary Data - Supplementary Data
