# Supplementary material for: Transcriptomic analysis comparing stay-green and senescent Sorghum bicolor lines identifies a role for proline biosynthesis in the stay-green trait
Source: J Exp Bot. 2015 Aug 28;66(22):7061–73. doi: 10.1093/jxb/erv405 (PMC4765785; doi:10.1093/jxb/erv405)
Supplement: Supplementary Data [file supp_erv405_jexbot155507_file001.pdf]

Transcriptomic analysis comparing stay-green and senescent *Sorghum bicolor* lines identifies a role for proline biosynthesis in the stay-green trait

Stephanie M. Johnson, Ian Cummins, Fei Ling Lim, Antoni R. Slabas, Marc R. Knight

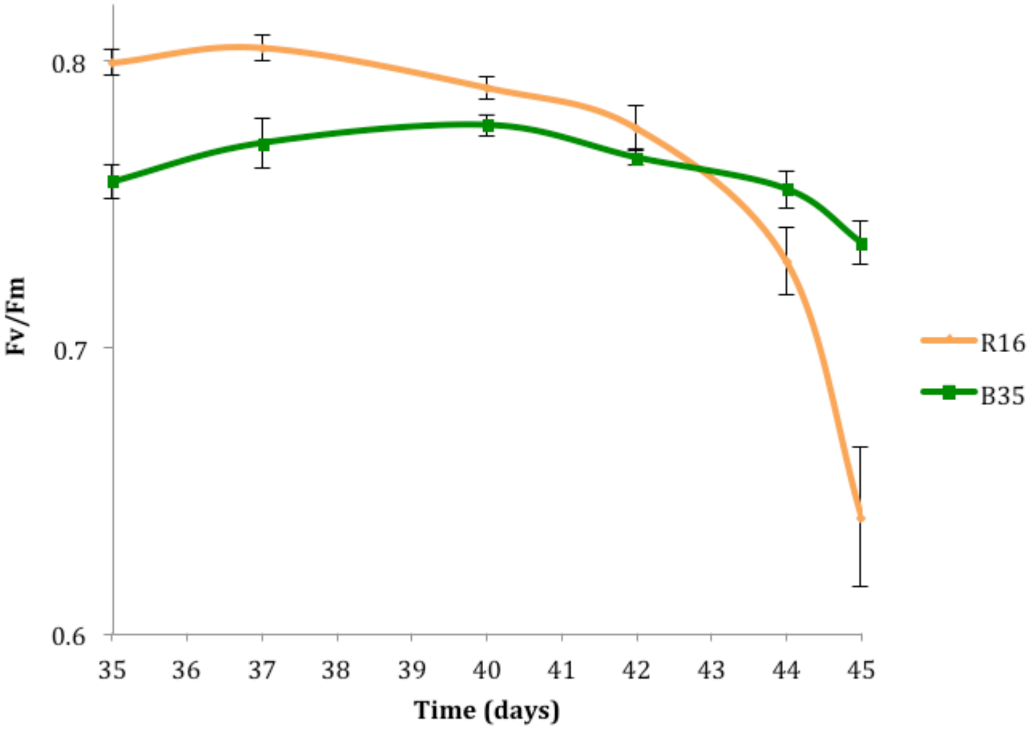

Figure S1 - Measurements of Fv/Fm in R16 and B35 from 35 DAS. Samples were taken for the microarray analysis at ~45 DAS. Error bars represent SEM, n = 8
